# Supplementary material for: Augmenting transcriptome assembly by combining de novo and genome-guided tools
Source: PeerJ. 2013 Aug 15;1:e133. doi: 10.7717/peerj.133 (PMC3746961; doi:10.7717/peerj.133)
Supplement: Supplemental Methods [file peerj-01-133-s003.pdf]

Supplementary Methods

Simulating RNA-seq reads

Parameter file used with Flux-simulator (TAIR10_nebulization.par)

$ grep exon TAIR10_GFF3_genes_transposons.gff | sed 's/Parent=/transcript_id "/g' | sed 's/$/"/g' > TAIR10_GFF3_genes_transposons.gff.gtf

```
## File locations
REF_FILE_NAME TAIR10_GFF3_genes_transposons.gff.gtf
GEN_DIR ./myGenome/

## Expression
NB_MOLECULES 5000000
TSS_MEAN NaN
POLYA_SCALE NaN
POLYA_SHAPE NaN

## Fragmentation
FRAGMENTATION YES
FRAG_SUBSTRATE DNA
FRAG_METHOD NB
FRAG_NB_LAMBDA 600

## RT parameters
RTRANSCRIPTION YES
RT_PRIMER PDT
RT_LOSSLESS YES
RT_MIN 100
RT_MAX 20000

## PCR / Filtering
PCR_DISTRIBUTION none
GC_MEAN    NaN
FILTERING YES
SIZE_DISTRIBUTION N(300,30)

## Sequencing
READ_NUMBER 4000000
READ_LENGTH 76
PAIRED_END YES
FASTA YES
UNIQUE_IDS YES
ERR_FILE 76
```

The sequence files in the "myGenome" directory as described above were downloaded from the following sites.
ftp://ftp.arabidopsis.org/home/tair/Sequences/whole_chromosomes/TAIR10_chr1.fas
ftp://ftp.arabidopsis.org/home/tair/Sequences/whole_chromosomes/TAIR10_chr2.fas

ftp://ftp.arabidopsis.org/home/tair/Sequences/whole_chromosomes/TAIR10_chr3.fas
ftp://ftp.arabidopsis.org/home/tair/Sequences/whole_chromosomes/TAIR10_chr4.fas
ftp://ftp.arabidopsis.org/home/tair/Sequences/whole_chromosomes/TAIR10_chr5.fas
ftp://ftp.arabidopsis.org/home/tair/Sequences/whole_chromosomes/TAIR10_chrC.fas
ftp://ftp.arabidopsis.org/home/tair/Sequences/whole_chromosomes/TAIR10_chrM.fas

Command to run Flux-simulator

/Apps/flux-simulator-1.1.1-20121119021549/bin/flux-simulator -p TAIR10_nebulization.par -x -l -s

Python script used to split interleaved fastq from Flux-simulator

export PYTHONPATH=/Apps/galaxy/lib:$PYTHONPATH
python /Apps/galaxy/tools/fastq/fastq_paired_unpaired-7ed81e36fc1c/tools/fastq/fastq_paired_unpaired.py sanger TAIR10_nebulization.fastq R1.fastq R2.fastq single.fastq

Read assembly

Command to run Trinity

perl /Apps/Trinity/trinityrnaseq_r2012-06-08/Trinity.pl --seqType fq --left ../R1.fastq --right ../R2.fastq --JM 2G --min_contig_length 75 --CPU 4 --bflyHeapSpaceMax 10G

Commands to run ABySS & Trans-ABySS

for k in {20..64}; do

mkdir k$k

/Apps/parallel/abyss-1.3/bin/abyss-pe -C k$k np=12 name='FS-20121119' k=$k lib='FS' FS='/FS-nightly-build_1.1.1-20121119021549/R1.fastq /FS-nightly-build_1.1.1-20121119021549/R2.fastq'

done

input_file:

FS-20121119 1.3.3 /FS-nightly-build_1.1.1-20121119021549/Trans-ABySS/Abyss_FS-20121119 Ath_sim

transcriptome.cfg:

[Ath_sim]

topdir: /FS-nightly-build_1.1.1-20121119021549/Trans-ABySS

reference: none

/Apps/parallel/trans-ABySS-v1.3.2/wrappers/trans-abyss.sh -c 192.168.1.10 -i input_file -0

Commands to run Velvet & Oases

/Apps/velvet_1.2.07/velveth dir 19,72,2 -shortPaired -fastq ../../TAIR10_nebulization.fastq

/Apps/velvet_1.2.07/velvetg dir_19 -ins_length 300 -exp_cov auto -read_trkg yes -unused_reads yes

/Apps/velvet_1.2.07/velvetg dir_21 -ins_length 300 -exp_cov auto -read_trkg yes -unused_reads yes

/Apps/velvet_1.2.07/velvetg dir_23 -ins_length 300 -exp_cov auto -read_trkg yes -unused_reads yes

/Apps/velvet_1.2.07/velvetg dir_25 -ins_length 300 -exp_cov auto -read_trkg yes -unused_reads yes

/Apps/velvet_1.2.07/velvetg dir_27 -ins_length 300 -exp_cov auto -read_trkg yes -unused_reads yes

/Apps/velvet_1.2.07/velvetg dir_29 -ins_length 300 -exp_cov auto -read_trkg yes -unused_reads yes

/Apps/velvet_1.2.07/velvetg dir_31 -ins_length 300 -exp_cov auto -read_trkg yes -unused_reads yes

/Apps/velvet_1.2.07/velvetg dir_33 -ins_length 300 -exp_cov auto -read_trkg yes -unused_reads yes

/Apps/velvet_1.2.07/velvetg dir_35 -ins_length 300 -exp_cov auto -read_trkg yes -unused_reads yes

/Apps/velvet_1.2.07/velvetg dir_37 -ins_length 300 -exp_cov auto -read_trkg yes -unused_reads yes

/Apps/velvet_1.2.07/velvetg dir_39 -ins_length 300 -exp_cov auto -read_trkg yes -unused_reads yes

/Apps/velvet_1.2.07/velvetg dir_41 -ins_length 300 -exp_cov auto -read_trkg yes -unused_reads yes

/Apps/velvet_1.2.07/velvetg dir_43 -ins_length 300 -exp_cov auto -read_trkg yes -unused_reads yes

/Apps/velvet_1.2.07/velvetg dir_45 -ins_length 300 -exp_cov auto -read_trkg yes -unused_reads yes

/Apps/velvet_1.2.07/velvetg dir_47 -ins_length 300 -exp_cov auto -read_trkg yes -unused_reads yes

/Apps/velvet_1.2.07/velvetg dir_49 -ins_length 300 -exp_cov auto -read_trkg yes -unused_reads yes

/Apps/velvet_1.2.07/velvetg dir_51 -ins_length 300 -exp_cov auto -read_trkg yes -unused_reads yes

/Apps/velvet_1.2.07/velvetg dir_53 -ins_length 300 -exp_cov auto -read_trkg yes -unused_reads yes

/Apps/velvet_1.2.07/velvetg dir_55 -ins_length 300 -exp_cov auto -read_trkg yes -unused_reads

yes

/Apps/velvet_1.2.07/velvetg dir_57 -ins_length 300 -exp_cov auto -read_trkg yes -unused_reads yes

/Apps/velvet_1.2.07/velvetg dir_59 -ins_length 300 -exp_cov auto -read_trkg yes -unused_reads yes

/Apps/velvet_1.2.07/velvetg dir_61 -ins_length 300 -exp_cov auto -read_trkg yes -unused_reads yes

/Apps/velvet_1.2.07/velvetg dir_63 -ins_length 300 -exp_cov auto -read_trkg yes -unused_reads yes

/Apps/velvet_1.2.07/velvetg dir_65 -ins_length 300 -exp_cov auto -read_trkg yes -unused_reads yes

/Apps/velvet_1.2.07/velvetg dir_67 -ins_length 300 -exp_cov auto -read_trkg yes -unused_reads yes

/Apps/velvet_1.2.07/velvetg dir_69 -ins_length 300 -exp_cov auto -read_trkg yes -unused_reads yes

/Apps/velvet_1.2.07/velvetg dir_71 -ins_length 300 -exp_cov auto -read_trkg yes -unused_reads yes

/Apps/oases_0.2.08/oases dir_19 -ins_length 300 -unused_reads yes -min_trans_lgth 76

/Apps/oases_0.2.08/oases dir_21 -ins_length 300 -unused_reads yes -min_trans_lgth 76

/Apps/oases_0.2.08/oases dir_23 -ins_length 300 -unused_reads yes -min_trans_lgth 76

/Apps/oases_0.2.08/oases dir_25 -ins_length 300 -unused_reads yes -min_trans_lgth 76

/Apps/oases_0.2.08/oases dir_27 -ins_length 300 -unused_reads yes -min_trans_lgth 76

/Apps/oases_0.2.08/oases dir_29 -ins_length 300 -unused_reads yes -min_trans_lgth 76

/Apps/oases_0.2.08/oases dir_31 -ins_length 300 -unused_reads yes -min_trans_lgth 76

/Apps/oases_0.2.08/oases dir_33 -ins_length 300 -unused_reads yes -min_trans_lgth 76

/Apps/oases_0.2.08/oases dir_35 -ins_length 300 -unused_reads yes -min_trans_lgth 76

/Apps/oases_0.2.08/oases dir_37 -ins_length 300 -unused_reads yes -min_trans_lgth 76

/Apps/oases_0.2.08/oases dir_39 -ins_length 300 -unused_reads yes -min_trans_lgth 76

/Apps/oases_0.2.08/oases dir_41 -ins_length 300 -unused_reads yes -min_trans_lgth 76

/Apps/oases_0.2.08/oases dir_43 -ins_length 300 -unused_reads yes -min_trans_lgth 76

/Apps/oases_0.2.08/oases dir_45 -ins_length 300 -unused_reads yes -min_trans_lgth 76

/Apps/oases_0.2.08/oases dir_47 -ins_length 300 -unused_reads yes -min_trans_lgth 76

/Apps/oases_0.2.08/oases dir_49 -ins_length 300 -unused_reads yes -min_trans_lgth 76

/Apps/oases_0.2.08/oases dir_51 -ins_length 300 -unused_reads yes -min_trans_lgth 76

/Apps/oases_0.2.08/oases dir_53 -ins_length 300 -unused_reads yes -min_trans_lgth 76

/Apps/oases_0.2.08/oases dir_55 -ins_length 300 -unused_reads yes -min_trans_lgth 76

/Apps/oases_0.2.08/oases dir_57 -ins_length 300 -unused_reads yes -min_trans_lgth 76

/Apps/oases_0.2.08/oases dir_59 -ins_length 300 -unused_reads yes -min_trans_lgth 76

/Apps/oases_0.2.08/oases dir_61 -ins_length 300 -unused_reads yes -min_trans_lgth 76

/Apps/oases_0.2.08/oases dir_63 -ins_length 300 -unused_reads yes -min_trans_lgth 76

/Apps/oases_0.2.08/oases dir_65 -ins_length 300 -unused_reads yes -min_trans_lgth 76

/Apps/oases_0.2.08/oases dir_67 -ins_length 300 -unused_reads yes -min_trans_lgth 76

/Apps/oases_0.2.08/oases dir_69 -ins_length 300 -unused_reads yes -min_trans_lgth 76

/Apps/oases_0.2.08/oases dir_71 -ins_length 300 -unused_reads yes -min_trans_lgth 76

/Apps/velvet_1.2.07/velveth MergedAssembly/ 27 -long dir_*/transcripts.fa

/Apps/velvet_1.2.07/velvetg MergedAssembly/ -read_trkg yes -conserveLong yes

/Apps/oases_0.2.08/oases MergedAssembly/ -min_trans_lgth 76 -merge yes

Config file used with SOAPdenovo-Trans

max_rd_len=76

[LIB]

avg_ins=300

reverse_seq=0

asm_flags=3

rank=1

q1=/Transcriptome_optimisation/FS-nightly-build_1.1.1-20121119021549/R1.fastq

q2=/Transcriptome_optimisation/FS-nightly-build_1.1.1-20121119021549/R2.fastq

Command to run SOAPdenovo-Trans

/Apps/SOAPdenovo-Trans/SOAPdenovo-Trans-31kmer all -s config -o FS-20121119 -L 76

Final output file: FS-20121119.contig

Commands to run Tophat1-Cufflinks

bowtie-build -f ../TAIR10_Ath_genome.fa_mod TAIR10genome

/Apps/tophat-2.0.4/tophat -r 148 -p 1 --bowtie1 ../TAIR10genome ../../R1.fastq ../../R2.fastq

/Apps/serial/cufflinks-2.0.0.Linux_x86_64/cufflinks tophat_out/accepted_hits.bam

/Apps/tophat-2.0.4/gtf_to_fasta transcripts.gtf TAIR10_Ath_genome.fa_mod transcripts.gtf.fasta

Commands to run Genome guided Trinity

/Apps/Trinity/trinityrnaseq_r2012-10-05/util/alignReads.pl --seqType fq --left ../../R1.fastq --right ../../R2.fastq --target ../../TAIR10_Ath_genome.fa_mod --aligner gsnap -- -t 6

samtools view gsnap_out/gsnap.coordSorted.bam > gsnap.coordSorted.sam

/Apps/Trinity/trinityrnaseq_r2012-10-05/util/prep_rnaseq_alignments_for_genome_assisted_assembly.pl --coord_sorted_SAM gsnap.coordSorted.sam -I 100000

find Dir_* -name "*reads" > read_files.list

/Apps/Trinity/trinityrnaseq_r2012-10-05/util/GG_write_trinity_cmds.pl --reads_list_file read_files.list --paired > trinity_GG.cmds

Modified the file trinity_GG.cmds to include the option min_contig_length 75.

/Apps/Trinity/trinityrnaseq_r2012-10-05/Inchworm/bin/ParaFly -c trinity_GG.cmds -CPU 6 -failed_cmds trinity_GG.cmds.failed -v

find Dir_* -name "*inity.fasta" -exec cat {} + | /Apps/Trinity/trinityrnaseq_r2012-10-05/util/inchworm_accession_incrementer.pl > Trinity_GG.fasta

Augmenting Trinity assembly with Tophat1-Cufflinks assembly

Blast to Top1Cuff transcripts:

/Apps/BLAST/blast+/2.2.26/ncbi-blast-2.2.26+/bin/makeblastdb -in /Transcriptome_optimisation/FS-nightly-build_1.1.1-20121119021549/PASA/Top1Cuff-r148-cleaned-L76.fasta -out Top1Cuff-db -dbtype nucl
/Apps/BLAST/blast+/2.2.26/ncbi-blast-2.2.26+/bin/blastn -task megablast -outfmt "6 qseqid qstart qend sseqid sstart send qlen slen evalue bitscore score length pident mismatch gaps" -db Top1Cuff-db -num_threads 12 -query /Transcriptome_optimisation/FS-nightly-build_1.1.1-20121119021549/PASA/Trinity-cleaned-L76.fasta -out Trinity-Top1Cuff.megablast
Subtracted the Trinity-hits from Top1Cuff transcripts
awk '{print $4"\t0\t"$8}' Trinity-Top1Cuff.megablast > uniq
awk '{if ($5<$6) print $4"\t"$5-1"\t"$6; else print $4"\t"$6-1"\t"$5}' Trinity-Top1Cuff.megablast > hits

/Apps/BEDTools/bin/sortBed -i uniq > uniq.sorted.BED
/Apps/BEDTools/bin/sortBed -i hits > hits.sorted.BED

/Apps/BEDTools/bin/subtractBed -a uniq.sorted.BED -b hits.sorted.BED | sort -u > SubtractedCufflinks.BED
awk '{print $1"\t"$2+1"\t"$3}' SubtractedCufflinks.BED > SubtractedCufflinks.gff3

Extracted fasta for subtracted portions:

bash extract_fasta_subsequence.sh SubtractedCufflinks.gff3 ../PASA/Top1Cuff-r148-cleaned-L76.fasta
sed 's/ bases /_/g' SubtractedCufflinks.gff3.fasta | sed 's/\.\./_/g' > T

mv T SubtractedCufflinks.gff3.fasta

Blasted subtracted.fasta + Trinity.fasta against MA for estimating recovery improvement:

```
/Apps/BLAST/blast+/2.2.26/ncbi-blast-2.2.26+/bin/blastn -task megablast -outfmt "6 qseqid qstart qend sseqid sstart send qlen slen evalue bitscore score length pident mismatch gaps" -db ../BLAST/MA-db -num_threads 12 -query SubtractedCufflinks.gff3.fasta -out SubtractedCufflinks.gff3.fasta.megablast
cat ../BLAST/Trinity-clean.megablast SubtractedCufflinks.gff3.fasta.megablast > Trinity-augmented.megablast
awk '{print $1"\t"$2"\t"$3"\t"$4"\t"$5"\t"$6}' Trinity-augmented.megablast > Trinity-augmented.megablast.6gff3
```

(remove nested and partial overlaps)

```
sed '/^$/d' Trinity-augmented.megablast.3.nR.pR.bed > t
mv t Trinity-augmented.megablast.3.nR.pR.bed
perl ../BLAST/expandRange.pl Trinity-augmented.megablast.3.nR.pR.bed
sort -u out > Trinity-augmented.megablast.3.nR.pR.bed.sort1
sort -k1,1 -k2n,2 Trinity-augmented.megablast.3.nR.pR.bed.sort1 > Trinity-augmented.megablast.3.nR.pR.bed.sort2
perl ../BLAST/collapseRange.pl Trinity-augmented.megablast.3.nR.pR.bed.sort2 > Trinity-augmented.megablast.3.nR.pR.bed.collapsed
```

(get recovery using Excel)

Redundancy removal using CDHit-EST

Command to run CDHit-EST

```
/Apps/cd-hit-4.5.4/cd-hit-est -i ModelAssembly-mod.fasta -o FS-MA-95 -c 0.95 -n 8 -r 1 -p 1 -g 1 -T 2 -d 40
```

Mapping of assembly to MA using Megablast

Command to run Megablast

```
/Apps/blast+/2.2.26/ncbi-blast-2.2.26+/bin/makeblastdb -in ModelAssembly-mod.fasta -out MA-db -dbtype nucl
```

```
/Apps/blast+/2.2.26/ncbi-blast-2.2.26+/bin/blastn -task megablast -outfmt "6 qseqid qstart qend sseqid sstart send qlen slen evalue bitscore score length pident mismatch gaps" -db MA-db -num_threads 12 -query Trinity.fasta.clean -out Trinity-clean.megablast
```

Script for removal of Nested Overlaps

Iterate the following until no Nested found (i.e. grep Nested temp | wc -l = 0)

perl removeNestedOverlaps.pl query.bed > temp

grep Nested temp | wc -l

grep -v Nested temp > query.bed

removeNestedOverlaps.pl:

```
$in=$ARGV[0];
open(IN,"<$in");
$first=1;
while (<IN>) {
	chomp $_;
	@W=split(/[\s]+/,$_);
	if (! $first) {
		if ($W[0] =~ /$prevqid/ && $prevqid =~ /$W[0]/) {
			if (($W[1] > $prevqs && $W[2] < $prevqe) || ($W[1] > $prevqs && $W[2]
== $prevqe) || ($W[1] == $prevqs && $W[2] < $prevqe)) {
				print "\n$_\tNested";
			}
			elsif (($prevqs > $W[1] && $prevqe < $W[2]) || ($prevqs > $W[1] &&
$W[2] == $prevqe) || ($W[1] == $prevqs && $prevqe < $W[2])) {
			print "\tNested\n$_";
		}
			else {
				print "\n$_";
			}
		}
		else {
			print "\n$_";
		}
	}
	else {
		print "\n$_";
	}
	$prevqid=$W[0];
	$prevqs=$W[1];
	$prevqe=$W[2];
	$first=0;

}
```

Script for removal of Partial Overlaps

The bed file resulting from running from the above script is taken through removal of partial overlaps that have a unique coverage of <10bp. The following lines are iterated until there are no partial overlaps (i.e. grep partial temp | wc -l = 0).

perl removePartialInsignificantOverlaps.pl query.bed > temp

grep Partial temp | wc -l

grep -v Partial temp > query.bed

removePartialInsignificantOverlaps.pl:

```
$in=$ARGV[0];
open(IN,"<$in");
$first=1;
while (<IN>) {
	chomp $_;
	@W= split(/[\s]+/,$_);
	if (! $first) {
		if ($W[0] =~ /$prevqid/ && $prevqid =~ /$W[0]/) {
			if ($W[1] > $prevqs && $W[2] > $prevqe) { # Partial
				if (($W[1] - $prevqs) < 10 && ($W[2]-$prevqe) > 10) {
					print "\tPartial\n$_";
				}
				elsif (($W[1] - $prevqs) > 10 && ($W[2] - $prevqe) < 10) {
					print "\n$_\tPartial";
				}
				elsif ((($W[1] - $prevqs) <= 10 && ($W[2] - $prevqe) <= 10)
||(($W[1] - $prevqs) >= 10 && ($W[2] - $prevqe) >= 10)) {
					print "\n$_";
				}
			}
			else {
				print "\n$_";
			}
		}
		else {
			print "\n$_";
		}
```

```
		}
		else {
			print "\n$_";
		}
		$prevqid=$W[0];
		$prevqs=$W[1];
		$prevqe=$W[2];
		$first=0;
}
print "\n";
```

Running the above two scripts on the query bed file corresponding to Megablast hits would maximize MA coverage of all queries, while running them on the subject bed file from Megablast hits would maximize the query coverage of all MA fragments.

R script for the box and whisker panel plot

```
df=read.table("/home/ganit/Documents/Transcriptome Assembly/testAll.Smax.trinityaugmented",header=TRUE,sep="\t")

library(plyr)

library(ggplot2)

plot_Data <- ddply(df, .(BinID, Assembler, L), mutate, med=median(Recovery),min=min(Recovery),max=max(Recovery),Q1=quantile(Recovery, 1/4), Q3=quantile(Recovery, 3/4), IQR=Q3-Q1, upper.limit=Q3+1.5*IQR, lower.limit=Q1-1.5*IQR)

p <- ggplot(data = plot_Data, aes(x = BinID))

p <- p + geom_boxplot(aes(lower = Q1, upper = Q3, middle = med, ymin = min, ymax = max), stat = "identity", colour="blue", outlier.colour = "black", outlier.shape = 16, outlier.size = 16)

p <- p + geom_point(data=plot_Data[plot_Data$Recovery > plot_Data$upper.limit | plot_Data$Recovery < plot_Data$lower.limit,], aes(y=Recovery))

p <- p + facet_grid(Assembler ~ L, scales="free", space="free")

p <- p + theme(plot.title = element_text("Pre CDHit-EST"),
```

```
        axis.text.x = element_text(angle = 90, hjust = 1, size = 8, colour = "grey50"),

        plot.title = element_text(face="bold", size=11),

        axis.title.x = element_text(face="bold", size=9),

        axis.title.y = element_text(face="bold", size=9, angle=90),

        panel.grid.major = element_blank(),

        panel.grid.minor = element_blank())

p <- p + scale_fill_hue(c=45, l=80)

p
```

snippet of testAll.Smax:

```
	Recovery	Assembler	L	BinID
1	90.7894736842	Trinity	76bp	B1
2	46.0526315789	Trinity	76bp	B1
3	57.8947368421	Trinity	76bp	B1
4	100	Trinity	76bp	B1
5	100	Trinity	76bp	B1
6	50	Trinity	76bp	B1
7	77.6315789474	Trinity	76bp	B1
8	98.6842105263	Trinity	76bp	B1
9	98.6842105263	Trinity	76bp	B1
```

R script for the assembly mapping statistics plot:

```
library(ggplot2)

library(plyr)

df=read.table("/home/ganit/Documents/Transcriptome
Assembly/lengthstats",header=TRUE,sep="\t")

df <- ddply(df, .(Bins, Assembler,Type), transform,
```

```
cum.perc = Reduce('+', list(Assemblies/2,cumsum(c(0,head(Assemblies,-1))))))

p <- ggplot(data = df, aes(x = Bins, y = Assemblies, fill = Mapping))
p <- p + geom_bar(stat = "identity", colour = "transparent")
p <- p + geom_text(aes(x = Bins, y = cum.perc, ymax = cum.perc, label = Numbers, hjust = 0.5), size=2, colour = "black", face = "bold")
p <- p + facet_grid(Type ~ Assembler, scales="free", space="free")
p <- p + theme(plot.title = element_text("Pre CDHit-EST"),
    axis.text.x = element_text(angle = 90, hjust = 1, size = 8, colour = "grey50"),
    plot.title = element_text(face="bold", size=11),
    axis.title.x = element_text(face="bold", size=9),
    axis.title.y = element_text(face="bold", size=9, angle=90),
    panel.grid.major = element_blank(),
    panel.grid.minor = element_blank())
p <- p + scale_fill_hue(c=45, l=80)
p
```

Snippet of lengthstats file:

Bins	Mapping	Assemblies	Assembler	Numbers	Type
0-0.01kb	>=90	73.4604939051	A_Trinity	11631	A_Pre_CDHit
0.01-0.02kb	>=90	84.570646596	A_Trinity	7416	A_Pre_CDHit
0.02-0.03kb	>=90	90.679933665	A_Trinity	2734	A_Pre_CDHit
0.03-0.04kb	>=90	88.9795918367	A_Trinity	872	A_Pre_CDHit
0.04-0.05kb	>=90	92.5	A_Trinity	555	A_Pre_CDHit
0.05-0.06kb	>=90	89.5918367347	A_Trinity	439	A_Pre_CDHit
0.06-1kb	>=90	93.6243936244	A_Trinity	1351	A_Pre_CDHit
1-2kb	>=90	97.3407977607	A_Trinity	1391	A_Pre_CDHit

2-5kb	>=90	99.0797546012	A_Trinity	323	A_Pre_CDHit
5-10.6kb	>=90	100	A_Trinity	8	A_Pre_CDHit
0-0.01kb	60-90	19.3961978147	A_Trinity	3071	A_Pre_CDHit
0.01-0.02kb	60-90	12.7494583191	A_Trinity	1118	A_Pre_CDHit
0.02-0.03kb	60-90	7.4626865672	A_Trinity	225	A_Pre_CDHit
0.03-0.04kb	60-90	9.2857142857	A_Trinity	91	A_Pre_CDHit
0.04-0.05kb	60-90	6	A_Trinity	36	A_Pre_CDHit
0.05-0.06kb	60-90	9.5918367347	A_Trinity	47	A_Pre_CDHit
0.06-1kb	60-90	5.8212058212	A_Trinity	84	A_Pre_CDHit
1-2kb	60-90	2.3093072078	A_Trinity	33	A_Pre_CDHit
2-5kb	60-90	0.6134969325	A_Trinity	2	A_Pre_CDHit
5-10.6kb	60-90	0	A_Trinity	0	A_Pre_CDHit
0-0.01kb	<60	7.1433082802	A_Trinity	1131	A_Pre_CDHit
0.01-0.02kb	<60	2.679895085	A_Trinity	235	A_Pre_CDHit

R script for heatmap analyses:

```
library(plyr)
library(reshape)
library(scales)
nba <- read.table("for_hm_larva_shared",header=TRUE,sep="\t")
nba$LARVA <- with(nba, reorder(LARVA, PNC))
library(ggplot2)
nba.m <- melt(nba)
nba.m <- ddply(nba.m, .(variable), transform,
    rescale = rescale(value))
(p <- ggplot(nba.m, aes(variable, LARVA)) + geom_tile(aes(fill = rescale),
```

```
    colour = "white") + scale_fill_gradient(low = "white",
    high = "steelblue"))
base_size <- 0.5
p + theme_grey(base_size = base_size) + labs(x = "",
    y = "") + scale_x_discrete(expand = c(0, 0)) +
    scale_y_discrete(expand = c(0, 0)) + theme(legend.position = "none",
    axis.ticks = element_blank(), axis.text.x = element_text(size = base_size *
        0.8, angle = 330, hjust = 0, colour = "grey50"))
        ggsave("larva_shared.png",plot=p,dpi=600)
```

R script for violin lot analyses (Supplementary Figure 2):

```
library(lattice)
test=read.table("/home/ganit/Documents/Transcriptome
Assembly/New.Old.binning.all",header=TRUE,sep="\t")
bwplot(New ~ Old | Assembler, data = test,
      panel = function(..., box.ratio = 0.1) {
          panel.violin(..., varwidth = TRUE, box.width = 0.5, layout = c(8, 8))
      })
par.settings = list(plot.symbol = list(pch = 21, col = "gray"),
box.rectangle = list(col ="blue"), box.umbrella = list(col = "black"))
```

Recovery of isoforms

```
cut -d : -f 1,3 --output-delimiter=_ TAIR10_nebulization.bed | awk '{print $4"\t"$2+1"\t"$3}' >
TAIR10_nebulization.3.gff3
awk '{print $1"\t"$2-1"\t"$3}' TAIR10_nebulization.3.gff3 > TAIR10_nebulization.3.bed
sed 's/"//g' TAIR10_GFF3_genes_transposons.gff_sorted.gtf | awk '{print $1"_"$10"\t"$4-1"\t"$5}'
> TAIR10_GFF3_genes_transposons.gff_sorted.gtf.3.bed
/Apps/BEDTools/bin/intersectBed -a TAIR10_nebulization.3.bed -b
TAIR10_GFF3_genes_transposons.gff_sorted.gtf.3.bed > Reads-Exons.intersection.bed
```

}

```
awk '{print $1"\t"$2+1"\t"$3}' Reads-Exons.intersection.bed > Reads-Exons.intersection.gff3
perl ../BLAST/expandRange.pl Reads-Exons.intersection.gff3
sort -u out > Reads-Exons.intersection.gff3.sort1
sort -k1,1 -k2n,2 Reads-Exons.intersection.gff3.sort1 > Reads-Exons.intersection.gff3.sort2
perl ../BLAST/collapseRange.pl Reads-Exons.intersection.gff3.sort2 > Reads-Exons.intersection.gff3.collapsed
awk '{SUM += $3-$2+1} END {print SUM}' Reads-Exons.intersection.gff3.collapsed   (16715077)

awk '{if ($3-$2+1 >= 28) print}' Reads-Exons.intersection.gff3.collapsed > Reads-Exons.intersection.gff3.collapsed.filteredBy28
sed 's/_AT[1-5MC]G[0-9]*\.[0-9]*//g' Reads-Exons.intersection.gff3.collapsed.filteredBy28 > Reads-Exons.intersection.gff3.collapsed.filteredBy28-mod
cat ../myGenome/Chr* > TAIR10.fa
sed 's/ CHROMOSOME.*$//g' TAIR10.fa | sed 's/chloroplast/ChrC/g' | sed 's/mitochondria/ChrM/g' > TAIR10-mod.fa
bash extract_fasta_subsequence.sh Reads-Exons.intersection.gff3.collapsed.filteredBy28-mod TAIR10-mod.fa


sed 's/ bases /_/g' Reads-Exons.intersection.gff3.collapsed.filteredBy28-mod.fasta | sed 's/\.\./_/g' > T
mv T Reads-Exons.intersection.gff3.collapsed.filteredBy28-mod.fasta

awk '{if (NR==1 && $0 ~/>/) {print$0;next}; if ($0 ~/^>/) {print"\n"$0;next} else {printf("%s",$0)}}' Reads-Exons.intersection.gff3.collapsed.filteredBy28-mod.fasta > T
mv T Reads-Exons.intersection.gff3.collapsed.filteredBy28-mod.fasta
```

creategrep.pl:

```
$in="exons_isoforms.csv";
open (IN,"<$in");
while (<IN>) {
	chomp $_;
	print "grep -A 1 -m 1 $_ Reads-Exons.intersection.gff3.collapsed.filteredBy28-mod.fasta'\n";
}
```

```
perl creategrep.pl | sed 's/fasta.*/fasta/g' > grep.sh
sort -u grep.sh > T
```

```
mv T grep.sh

sh grep.sh | sed '/^--$/d' > isoforms-exons.fasta


/Apps/BLAST/blast+/2.2.26/ncbi-blast-2.2.26+/bin/makeblastdb -in isoforms-exons.fasta -out IsoExons-db -dbtype nucl


/Apps/BLAST/blast+/2.2.26/ncbi-blast-2.2.26+/bin/blastn -task megablast -outfmt "6 qseqid qstart qend sseqid sstart send qlen slen evalue bitscore score length pident mismatch gaps" -db IsoExons-db -num_threads 12 -query ../BLAST/Trinity.fasta.clean -out IsoExon-Trinity-clean.megablast

/Apps/BLAST/blast+/2.2.26/ncbi-blast-2.2.26+/bin/blastn -task megablast -outfmt "6 qseqid qstart qend sseqid sstart send qlen slen evalue bitscore score length pident mismatch gaps" -db IsoExons-db -num_threads 12 -query ../BLAST/FS-20121119-contigs.fa.clean -out IsoExon-TA-clean.megablast

/Apps/BLAST/blast+/2.2.26/ncbi-blast-2.2.26+/bin/blastn -task megablast -outfmt "6 qseqid qstart qend sseqid sstart send qlen slen evalue bitscore score length pident mismatch gaps" -db IsoExons-db -num_threads 12 -query ../BLAST/transcripts.fa.clean -out IsoExon-Oases76-clean.megablast

/Apps/BLAST/blast+/2.2.26/ncbi-blast-2.2.26+/bin/blastn -task megablast -outfmt "6 qseqid qstart qend sseqid sstart send qlen slen evalue bitscore score length pident mismatch gaps" -db IsoExons-db -num_threads 12 -query ../BLAST/FS-20121119.contig.clean -out IsoExon-SoapContig76-clean.megablast

/Apps/BLAST/blast+/2.2.26/ncbi-blast-2.2.26+/bin/blastn -task megablast -outfmt "6 qseqid qstart qend sseqid sstart send qlen slen evalue bitscore score length pident mismatch gaps" -db IsoExons-db -num_threads 12 -query ../BLAST/transcripts.gtf.fasta-mod.clean -out IsoExon-Top1Cuff-clean.megablast

/Apps/BLAST/blast+/2.2.26/ncbi-blast-2.2.26+/bin/blastn -task megablast -outfmt "6 qseqid qstart qend sseqid sstart send qlen slen evalue bitscore score length pident mismatch gaps" -db IsoExons-db -num_threads 12 -query ../BLAST/Trinity_GG.fasta.clean -out IsoExon-GGTrinity76-clean.megablast
```
