# Supplementary material for: Augmenting transcriptome assembly by combining de novo and genome-guided tools
Source: PeerJ. 2013 Aug 15;1:e133. doi: 10.7717/peerj.133 (PMC3746961; doi:10.7717/peerj.133)
Supplement: Table S1 [file peerj-01-133-s004.docx]

Supplementary Table 1: MA Recovery with simulations using altered parameters

|  | **# of MAs Recovered (%)** | | **Cumulative Length of MAs Recovered (%)** | |
| --- | --- | --- | --- | --- |
| **Assembler** | **76bp_100M** | **100bp_4M** | **76bp_100M** | **100bp_4M** |
| **Trinity** | 72.06 | 64.08 | 95.08 | 95.17 |
| **TransABySS** | 41.00 | 28.66 | 83.79 | 83.66 |
| **Oases** | 51.90 | 45.81 | 93.85 | 91.62 |
| **SOAPdenovo-Trans** | 66.90 | 55.46 | 92.40 | 91.15 |
| **Tophat1-Cufflinks** | 58.06 | 47.84 | 97.09 | 97.44 |
| **Genome guided Trinity** | 70.26 | 60.19 | 95.37 | 95.59 |

100 million 76bp and 4 million 100bp reads were simulated, and assembled using all six assemblers. The number of MAs (%) and their cumulative lengths recovered (%) are reported here.
